# Prolonged Effects of Silver Nanoparticles on p53/p21 Pathway-Mediated Proliferation, DNA Damage Response, and Methylation Parameters in HT22 Hippocampal Neuronal Cells

**DOI:** 10.1007/s12035-016-9688-6

**Published:** 2016-02-03

**Authors:** Jennifer Mytych, Jacek Zebrowski, Anna Lewinska, Maciej Wnuk

**Affiliations:** 1 Department of Genetics, University of Rzeszow, Rejtana 16C, 35-959 Rzeszow, Poland; 2 Department of Plant Physiology, University of Rzeszow, Werynia 502, 36-100 Kolbuszowa, Poland; 3 Department of Biochemistry and Cell Biology, University of Rzeszow, Zelwerowicza 4, 35-601 Rzeszow, Poland

**Keywords:** Silver nanoparticles, HT22 cells, DNA methylation, DNMTs, Oxidative stress

## Abstract

It is widely accepted that silver nanoparticles (AgNPs) are toxic to biological systems. However, little is known about their actions at molecular level and the cytophysiological effects after AgNP removal. As nanoparticles are suggested a promising tool to transport drugs to the brain for use in neurological conditions, we used HT22 mouse hippocampal neuronal cells as a model to study AgNP-mediated effects after their removal from the cell culture medium. We selected a relatively low concentration of AgNPs, 5 μg/ml, treated the cells for 48 h, and evaluated AgNP-induced cytophysiological effects after 96 h of AgNP removal. AgNP removal did not result in cytotoxicity. In contrast, AgNPs modulated HT22 cell cycle and proliferation and induced oxidative stress and 53BP1 recruitment, which were accompanied by elevated levels of p53 and p21. AgNP-associated diminution in lamin B1 pools did not significantly affect the structure of the nucleus. No disruption in F-actin dynamics was observed upon AgNP treatment. Moreover, we showed for the first time that AgNPs stimulated changes in DNA methylation: the augmentation in 5-methylcytosine (5-mC) and DNMT1, DNMT2, DNMT3a, and DNMT3b levels were observed. The upregulation of DNMT2 may be a part of cellular stress response to AgNP treatment. Taken together, AgNP removal resulted in p53/p21-mediated inhibition of cell proliferation, oxidant-based DNA damage response, and changes in DNA methylation patterns, which suggests that more attention should be paid to the possible outcomes in individuals exposed to nano-sized biomaterials.

## Introduction

Silver-nanoparticle-based materials with potent antimicrobial activities and unique physico-chemical properties are widely used in electronics, biosensing, clothing, food industry, paints, sunscreens, cosmetics, and medical devices [1–3]. Due to numerous applications of silver nanoparticles (AgNPs) in biomedical sciences, a comprehensive analysis of AgNP effects both short- and long-term actions on biological systems is needed.

It has been repeatedly reported that AgNPs induced toxicity both in vitro and in vivo [4–7]. AgNPs affected a plethora of normal and cancer cell lines and AgNP toxicity was accompanied by oxidative stress: increased production of reactive oxygen species (ROS) and/or glutathione depletion and decreased activity of antioxidant enzymes, which may contribute to decreased proliferation, genotoxic events, and/or apoptosis [4, 5, 8–14]. Despite some controversies on the mechanisms of action of AgNPs [14–16], AgNP toxicity is suggested to be independent of the toxicity of silver ions [12]. AgNPs and silver ions were also shown to trigger two different cellular responses as estimated using quantitative proteomics [17].

AgNPs may affect the global gene expression [18–20], but the mechanisms underlying such regulation are largely unknown. After treatment of human lung A549 and cervical HeLa cancer cells with AgNPs, the expression profiles of more than 1000 genes were modulated, e.g., encoding metallothioneins, heat shock proteins, and histone proteins [18, 19]. Moreover, AgNP-mediated defective DNA repair, proliferation arrest, and inflammatory response in IMR-90 human lung fibroblasts and U251 glioblastoma cells may be a consequence of AgNP-induced changes in the gene expression patterns, such as the upregulation of DNA damage response genes (*GADD45*) and the downregulation of cell cycle progression genes encoding cyclin B and cyclin E, and involved in DNA damage repair (*XRCC1*, *XRCC3*, *FEN1*, *RAD51C*, *RPA1*) [20]. AgNPs, when used at non-cytotoxic concentrations (<0.5 μg/ml), may also promote some adverse effects by affecting the expression of genes associated with cell cycle progression and apoptosis in human hepatoma cells (HepG2) [7]. Data on the mechanisms of AgNP-mediated regulation of the gene expression and protein translation are limited [21]. More recently, AgNP-induced *MT1F* (metallothionein 1 F) and *TRIB3* (tribbles homolog 3) expressions have been reported to be regulated by miR-219-5p in Jurkat T cells [21], which suggest the involvement of an epigenetic mechanism.

Little is known on prolonged effects of low, non-cytotoxic doses of AgNPs in the brain tissue. AgNP-induced dopaminergic neurotoxicity has been revealed in PC-12 rat neuronal cell line [22, 23]. AgNPs also caused a significant stress response in the growing human embryonic neural precursor cells (HNPCs) by simultaneously affecting cell proliferation and apoptotic cell death [24]. AgNP-mediated calcium dysregulation and reactive oxygen species (ROS) formation–based response have been observed in a mixed primary cell model (neurons, astrocytes, and a minor proportion of oligodendrocytes) [25]. AgNP-induced calcium imbalance, destabilization of mitochondrial function, and ROS production have also been reported in primary cultures of cerebellar granule cells [26]. More recently, sublethal concentrations of AgNPs have been found to disrupt actin dynamics in cultured adult neural stem cells [27]. However, data on the cytophysiological effects after AgNP removal from biological systems are lacking, especially AgNP-mediated effects on neural cell epigenome.

HT22 cells are considered as a sensitive model for monitoring cellular responses to oxidative stress due to the lack of ionotropic glutamate receptors [28] and are widely used to study the mechanisms of neurotoxicity and to search for neuroprotective compounds [29–31]. In the present study, we used HT22 mouse hippocampal neuronal cell line to evaluate prolonged effects of low concentration of AgNPs (5 μg/ml); especially, we were interested if cell proliferation, redox state, DNA damage response, and methylation parameters may be affected after AgNP removal.

## Materials and Methods

### Chemicals

Dihydroethidium and MitoSOX™ were purchased from Molecular Probes (Leiden, Netherlands) and phosphate-buffered saline (PBS) was obtained from (Gibco, Invitrogen Corporation, Grand Island, NY, USA). All other reagents, if not mentioned otherwise, were purchased from Sigma (Poznan, Poland) and were of analytical grade.

### Nanoparticle Size and Zeta Potential Measurements

Silver nanoparticles (AgNPs), <100-nm particle size (TEM; 758329, Sigma, Poznan, Poland), were characterized. Both particle size and the zeta potential of AgNPs dispersed in water were measured using ZetaSizer Nano ZS (Mavern Instruments, Mavern, UK) equipped with a 633-nm laser. The AgNP concentration and pH were adjusted to values characteristic for suspension of the particles in culture medium used. The dispersion was measured at 25 °C. The particle size distribution was assessed in a dynamic light scattering (DLS) mode on the base of a correlation function analysis for scattering angle of 173° (non-invasive back-scatter technology). The refraction index for silver material was assumed equal to 0.135. Prior to measurements, the samples were sonicated for 30 min. Five replicates were performed per measurement. The zeta potential of AgNPs in the medium (pH = 7.2) was assessed on the basis of Laser Doppler Velocimetry (LDV) taking into account their electrophoretic mobility. The Smoluchowski approximation was chosen for zeta potential evaluation. Three replicates were performed per measurement, each at hundred runs.

### Nanoparticle Agglomeration Analysis

Atomic force microscopy (AFM) was used to elucidate the tendency of AgNPs to agglomerate in DMEM culture medium and the susceptibility of serum components to agglomerate in the presence of AgNPs. The appropriate suspensions were deposited on atomic flat mica substrate (V1 grade, Ted Pella Inc., USA) and allowed to dry under N_2_ stream. AFM height sensor images were collected in Peak Force Tapping mode using Bioscope Catalyst II atomic force microscope equipped with Nanoscope V controller (Veeco, Santa Barbara, CA, USA). AFM topography imaging was performed in the air using Bruker silicon scanasyst-fluid + probes. Images were processed and analyzed for nanoparticle height by means of Nanoscope Analysis (v. 1.40 R3sr5, Bruker) software.

### Protein Corona Formation Analysis

The ability of the serum proteins to bind AgNPs was evaluated using sodium dodecyl sulfate polyacrylamide gel electrophoresis (SDS-PAGE) and Coomassie staining. Briefly, DMEM medium containing 10 % FBS was incubated with 5 μg/ml AgNPs at 37 °C for 48 h. AgNP-treated medium was then centrifuged (15,000 rpm, 15 min, 4 °C), washed three times with ice-cold PBS (15,000 rpm, 15 min, 4 °C), and added with 25 μl of PBS and 25 μl of 2× Laemmli buffer. The samples were then vigorously mixed, boiled at 95 °C for 5 min, and centrifuged (15,000 rpm, 15 min). The supernatants were collected and resolved on 10 % SDS-PAGE. The gel was stained using 0.25 % Coomassie Brilliant Blue R-250 in 45 % methanol and 10 % acetic acid for 30 min, washed twice with water for 10 min, and de-stained using 45 % methanol and 10 % acetic acid solution for 1 h.

### Cell Culture

HT22 mouse hippocampal neuronal cell line was a generous gift from Prof. Michal Wozniak and Dr. Magdalena Gorska (Medical University of Gdansk, Gdansk, Poland). Cells (3000 cells/cm^2^) were cultured at 37 °C in Dulbecco’s modified Eagle’s medium (DMEM) supplemented with 10 % fetal calf serum (FCS) and antibiotic and antimycotic mix solution (100 U/ml penicillin, 0.1 mg/ml streptomycin, and 0.25 μg/ml amphotericin B) in a humidified atmosphere in the presence of 5 % CO_2_ until they reached confluence. Typically, cells were passaged by trypsinization and maintained in DMEM. The freshly prepared stock solution of AgNPs (10 mg/ml in sterile PBS) was added to HT22 cells to obtain final concentrations ranging from 1 to 20 μg/ml.

### Cytotoxicity, Cell Proliferation, and Cell Cycle Analysis

After 48-h treatment with AgNPs (1–20 μg/ml), cytotoxic/cytostatic potential was estimated using MTT assay [32], cell proliferation was established using bromodeoxyuridine (BrdU) incorporation assay [33], and DNA-content-based cell cycle analysis was conducted using imaging cytometry [34]. A concentration reflecting IC_50_ value (MTT assay), 5 μg/ml, was selected to study prolonged effects of AgNPs. Briefly, HT22 cells were incubated with 5 μg/ml AgNPs for 48 h, washed three times with PBS, and cultured in AgNP-free medium for up to 144 h (96 h after AgNP removal). Every 48 h, the cells were washed three times with PBS and the medium was replaced by a fresh one. Cytotoxicity, cell cycle, and cell proliferation (DNA synthesis) were then evaluated. Cytotoxicity was assayed using acridine orange-ethidium bromide staining [35]. HT22 cells were washed twice with PBS, and a mixture of acridine orange (100 μg/ml in PBS) and ethidium bromide (100 μg/ml in PBS) at a volume ratio of 1:1 was added to the cells, which were then analyzed with an Olympus BX61 fluorescence microscope equipped with a DP72 CCD camera and Olympus CellF software. Live/dead cell analysis was conducted according to the principle that acridine orange stained live cells green, while ethidium bromide stained dead cells red to orange. A total of 200 cells were counted.

For DNA-content-based cell cycle analysis [34], HT22 cells were stained with Hoechst 33342 (2.5 μg/ml) and digital cell images were captured with an In Cell Analyzer 2000 (GE Healthcare, UK) equipped with a high-performance CCD camera. DNA content of a total of 200 cells was analyzed using ImageJ software and DNA Cell Cycle plug-in from MBF Collection (http://imagej.net/plugins/mbf/mbf-plugins.zip).

For DNA synthesis, BrdU assay was used [33]. BrdU was added to the medium (10 μM) for 24 h and was detected using primary antibody against BrdU (Becton Dickinson, Poland). A total of 200 cells were analyzed under a fluorescence microscope, and the % of BrdU positive cells was calculated. Moreover, cells were counted using a Bürker chamber.

### Silver Ion and Serum Effects

The effects of AgNO_3_ on cell viability were also evaluated. HT22 cells were cultured with AgNO_3_ (1–20 μg/ml) for 48 h and MTT assay was conducted. Moreover, to evaluate the effect of silver ion release from AgNPs, DMEM medium was pre-incubated with AgNPs (5 μg/ml) for 48 h, centrifuged (15,000 rpm, 15 min), and used as a cell culture medium. HT22 cells were also cultured in a serum-free medium in the presence of 5 μg/ml AgNPs to establish serum-associated effect on AgNP-mediated cell viability (MTT assay).

### Morphology, F-actin, and Nucleus Labeling

Cell morphology was monitored using an Olympus BX71 inverted microscope equipped with a DP72 CCD camera and a computer image analysis system CellB. Intracellular localization of AgNPs after 48-h treatment and after AgNP removal was evaluated using Nomarski microscopy. To analyze AgNP-mediated changes in nucleus, HT22 cells were fixed [32] and DNA was visualized using Hoechst 33342 staining. F-actin was stained using Alexa Fluor® 488 Phalloidin (a high-affinity filamentous actin, F-actin, probe conjugated to green fluorescent Alexa Fluor® 488 dye) according to manufacturer’s instructions (Life Technologies). Additionally, lamin B1 was immunodetected using lamin B1 antibody (1:100, Life Technologies) and secondary antibody conjugated with Texas red (1:1000, Life Technologies). Digital cell images were captured with an Olympus BX61 fluorescence microscope equipped with a DP72 CCD camera and Olympus CellF software. To analyze nuclear lamin B1 content, ImageJ software http://rsbweb.nih.gov/ij/ was used. We evaluated the integrated fluorescence density (red channel), which is the sum of all pixel values within the marked area of each nucleus analyzed and equivalent to the product of area and mean gray value. The integrated fluorescence density is presented in relative fluorescence units (RFUs). A total of 2000 cells were analyzed.

### Oxidative Stress Parameters

Intracellular reactive oxygen species (ROS) production and superoxide production, both total and mitochondrial, were measured using 2′,7′-dichlorodihydrofluorescein diacetate (H_2_DCF-DA), dihydroethidium, and MitoSOX^TM^, respectively, as described elsewhere [32].

### 53BP1 Immunostaining

For 53BP1 immunostaining, interphase nuclei were used. HT22 cells were fixed with 3.7 % formaldehyde containing 0.1 % Triton X-100 in PBS for 20 min and incubated with 1 % bovine serum albumin (BSA) in phosphate-buffered saline containing 0.25 % Triton X-100 (PBST) at room temperature for 30 min. After washing with PBST, the cells were incubated with a rabbit polyclonal antibody against 53BP1 (Novus Biologicals, Poland; diluted 1:200 in PBST–BSA (PBST containing 1 % BSA)) overnight at 4 °C, and with a FITC-conjugated, secondary polyclonal antibody against rabbit IgG (BD Biosciences, Germany; diluted 1:200 in PBST-BSA) at room temperature for 1 h. Nuclei were visualized with Hoechst 33342. Digital cell images were captured with an In Cell Analyzer 2000 (GE Healthcare, UK) equipped with a high-performance CCD camera. The cells with 0, 1–3, 4–9, and more than 10 53BP1 foci (48-h treatment with 1–20 μg/ml AgNPs) or cells with 0, 1, 2, and 3 53BP1 foci (96 h after AgNP removal) were scored (%).

### Western Blotting

Whole cell protein extracts were prepared according to Mytych et al. [32]. Polivinylidene difluoride (PVDF) membrane was incubated with one of the primary antibodies: anti-p21 (1:100), anti-p53 (1:1000), anti-lamin B1 (1:1000), anti-DNMT2 (1:500), and anti-β-actin (1:1000) (Abcam, Thermo Scientific and Novus Biologicals) and secondary antibody conjugated with HRP (1:80,000; Sigma). The respective proteins were detected using the ECL Plus system (GE Healthcare), according to the manufacturer’s instructions. The data represent the relative density normalized to β-actin [32].

### RNA Status

RNA was isolated using RNeasy Mini Kit (Qiagen, USA). RNA chip electrophoresis was performed with Experion™ Automated Electrophoresis System and Experion™ RNA Std-Sens Analysis Kit (Biorad, Germany). Total RNA level (pg) was calculated per cell and RNA integrity as a 28S/18S rRNA ratio [33].

### Methylation Parameters

The levels of 5-methylcytosine (5-mC), and DNA methyltransferases 1, 3a, and 3b (DNMT1, DNMT3a and DNMT3b) were measured using MethylFlash^TM^ Methylated DNA Quantification Kit, EpiQuik^TM^ DNMT1 Assay Kit, EpiQuik^TM^ DNMT3a Assay Kit, and EpiQuik^TM^ DNMT3b Assay Kit, respectively, (Epigentek, Farmingdale, NY, USA) according to the manufacturer’s instructions. The calculations were made on the basis of the standard curves obtained for positive control solutions and 5-mC content is presented as nanograms of 5-mC, and DNMT1, DNMT3a, and DNMT3b levels as ng/mg protein.

### Statistical Analysis

The results represent the mean ± SD from at least three independent experiments. The obtained data conform the ANOVA assumptions as evaluated using Shapiro–Wilk normality test and Levene test for the equality of variances. Statistical significance was assessed by one-way ANOVA using GraphPad Prism 5, with Dunnett’s multiple comparison test.

## Results

**Fig. 1:**
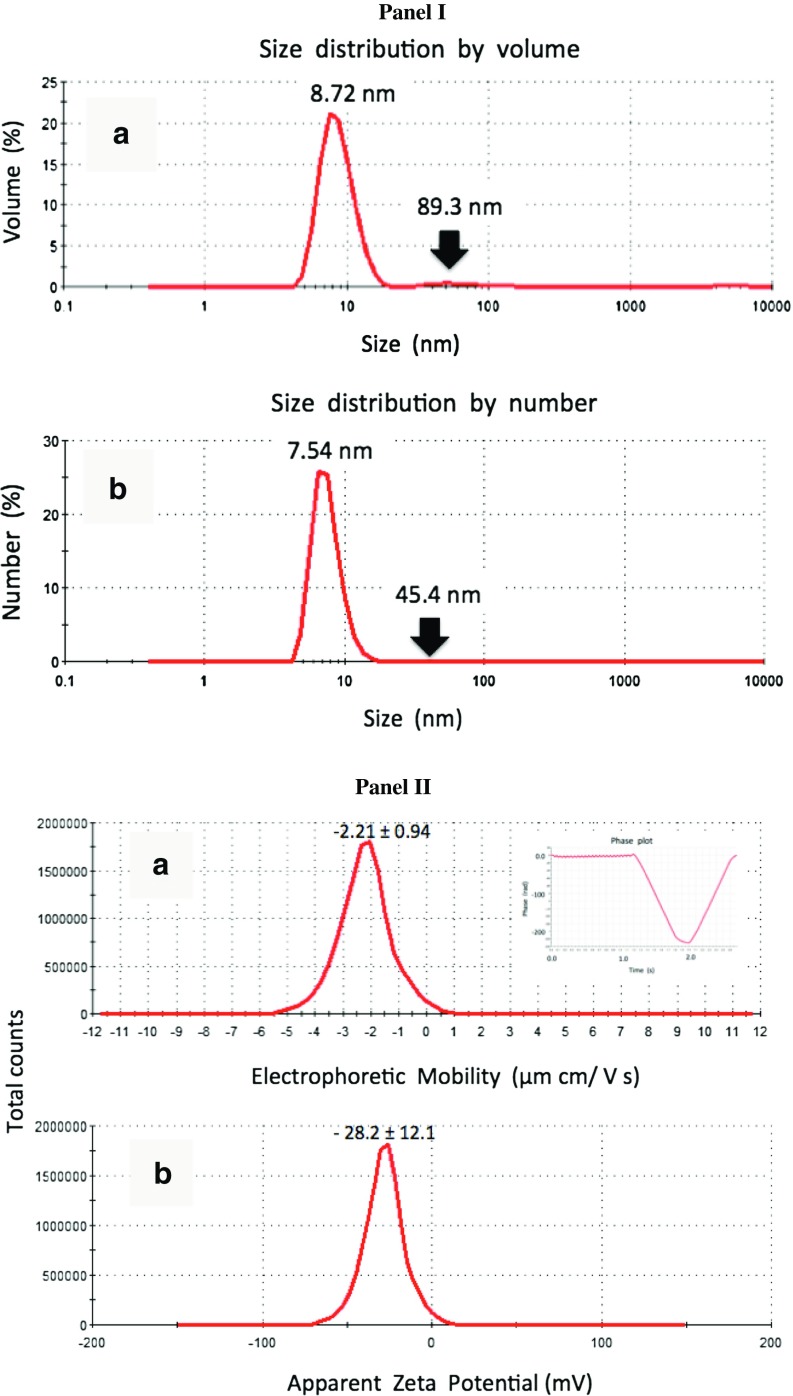

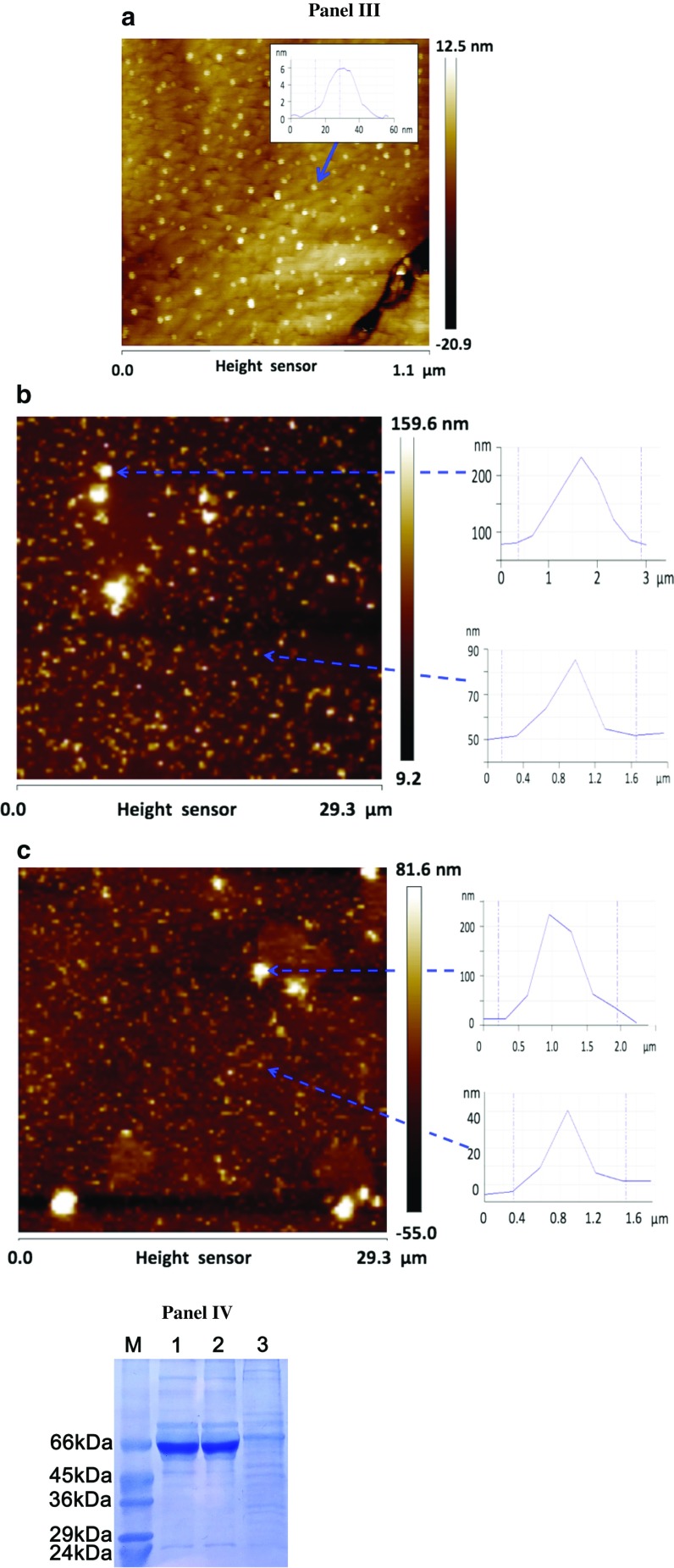
AgNP characterization. *Panel I*: Representative distribution patterns of silver nanoparticle size by volume (**a**) and number (**b**). The measurements were performed using dynamic light scattering (DLS) method. The size value corresponds to a peak position. *Black arrows* indicate low, broad peaks corresponding to agglomerated particles. *Panel II*: Representative electrophoretic mobility (**a**) and zeta potential (**b**) distributions of silver nanoparticles obtained for hundred runs. The inset in **a** shows a high quality phase plot generated for this dispersion system using Laser Doppler Velocimetry (LDV). The results are presented as mean ± SD. *Panel III*: Representative atomic force microscopy (AFM) imaging of DMEM medium with and without serum and with and without AgNPs incubated for 48 h and deposited on atomic flat mica surface. (**a**) AgNPs (5 μg/ml) suspended in a serum-free DMEM. The particles were of size from a few to about 20 nm (the height profile used for particle size measurements is given in the inset) and did not agglomerate. (**b**) DMEM with serum in the absence of AgNPs and (**c**) DMEM with serum in the presence of AgNPs (5 μg/ml). The globular complexes of approximately 10 nm in size were typically observed, whereas the agglomerates of the size above 100 nm were rarely observed. The presence of AgNPs did not substantially alter the particle size distribution. Height sensor images were obtained in the Peak Force mode*. Arrows* indicate nano-objects subjected to height profile analysis. *Panel IV*: The ability of serum proteins to bind to the surface of AgNPs to form coating (the protein corona) estimated using SDS-PAGE. Mild serum protein enrichment on the surface on AgNPs was observed. *Lane M*: protein marker, *Lane 1*: DMEM medium with 10 % FBS, *Lane 2*: 10 % FBS in PBS, *Lane 3*: DMEM medium with 10 % FBS incubated with AgNPs (5 μg/ml) for 48 h (see “Materials and Methods” section for more details)

As AgNPs were commercially purchased, we initially analyzed the AgNP size, dispersion stability, tendency to agglomerate in the cell culture medium, and protein corona formation (Fig. 1).

The hydrodynamic size distribution analysis of AgNPs at a concentration of 5 μg/ml showed a mean value of size equal to 8.05 ± 0.88 nm. The polydispersity index of the system was 0.66 and thus below critical value of 0.7, indicating suitability of the samples for DLS-based analysis and mono-size particle dispersion assay. Typical size distribution patterns by volume (a) and number (b) are shown in Fig. 1 in Panel I. A prominent peak at about 8.7 nm and very low, broad peak around value of 90 nm were observed (Fig. 1, Panel I, a). Volumetric fraction of the particles above 20 nm, which may be considered as AgNP agglomerates, was 3.2 %. However, the transformation of these data into size distribution by number (Fig. 1, Panel I, b) indicated that almost 100 % of particles in sample are of the size around 7.54 ± 1.77 nm, while localization of the peak corresponding to the fraction of potentially clustered particles was shifted to 45.4 ± 16.2 nm. This confirms that the particle fraction, which may be considered as agglomerates, is rather marginal. The dynamic light scattering (DLS) analysis showed variability of AgNPs in size ranging from approximately 3 nm to approximately 15 nm. The zeta potential of the AgNP dispersion showed a negative value and was equal to about −28 mV (Fig. 1, Panel II, b). This value is close to a critical value of −30 mV predicting potential physical stability of the suspension. Thus, this indicates a low tendency of AgNPs to aggregation because of the occurrence of the surface electrical charge high enough to prevent particle interactions. These data are consistent with estimation of the AgNP size in the same dispersion system, which suggest the presence of a very low fraction of agglomerated particles exceeding size of 20 nm. Moreover, AgNPs were characterized using atomic force microscopy (AFM; Fig. 1, Panel III). The particles were quite uniformly distributed and did not show a tendency to agglomerate in the DMEM medium. The size of the particles ranging from a few to 20 nm was consistent with that determined hydrodynamically using ZetaSizer for water suspensions and with the size of AgNPs observed by atomic force microscopy when suspended in water [36]. To elucidate whether the presence of AgNPs in serum suspension substantially favored the formation of protein agglomerates as a result of short-term incubation (48 h), we compared the AFM images of the DMEM and serum suspensions with and without AgNPs. In the suspension without serum, the nanostructures exhibiting the size from 20 to a few tens of nanometers were dominated (Fig. 1, Panel IIIa). The fraction of larger particles, around a hundred and more, occurred at much lower frequency. Representative height profiles are included on the right of the images (Fig. 1, Panel IIIa). Similar particle size distribution was observed after 48-h incubation without and with 5 μg/ml AgNPs (Fig. 1, Panel IIIb and c). Two groups of particles could be distinguished and both fractions were represented by the particles of similar size compared to the suspension without AgNPs. Therefore, there is no evidence that short-term incubation with AgNPs in culture medium considerably affected the tendency to agglomeration. The ability of serum proteins to bind to the surface of AgNPs was also evaluated (Fig. 1, Panel IV). Serum protein enrichment was observed only when the total medium volume used (15 ml) was concentrated and subjected to protein corona analysis (Fig. 1, Panel IV) after 48-h treatment with 5 μg/ml AgNPs.

**Fig. 2:**
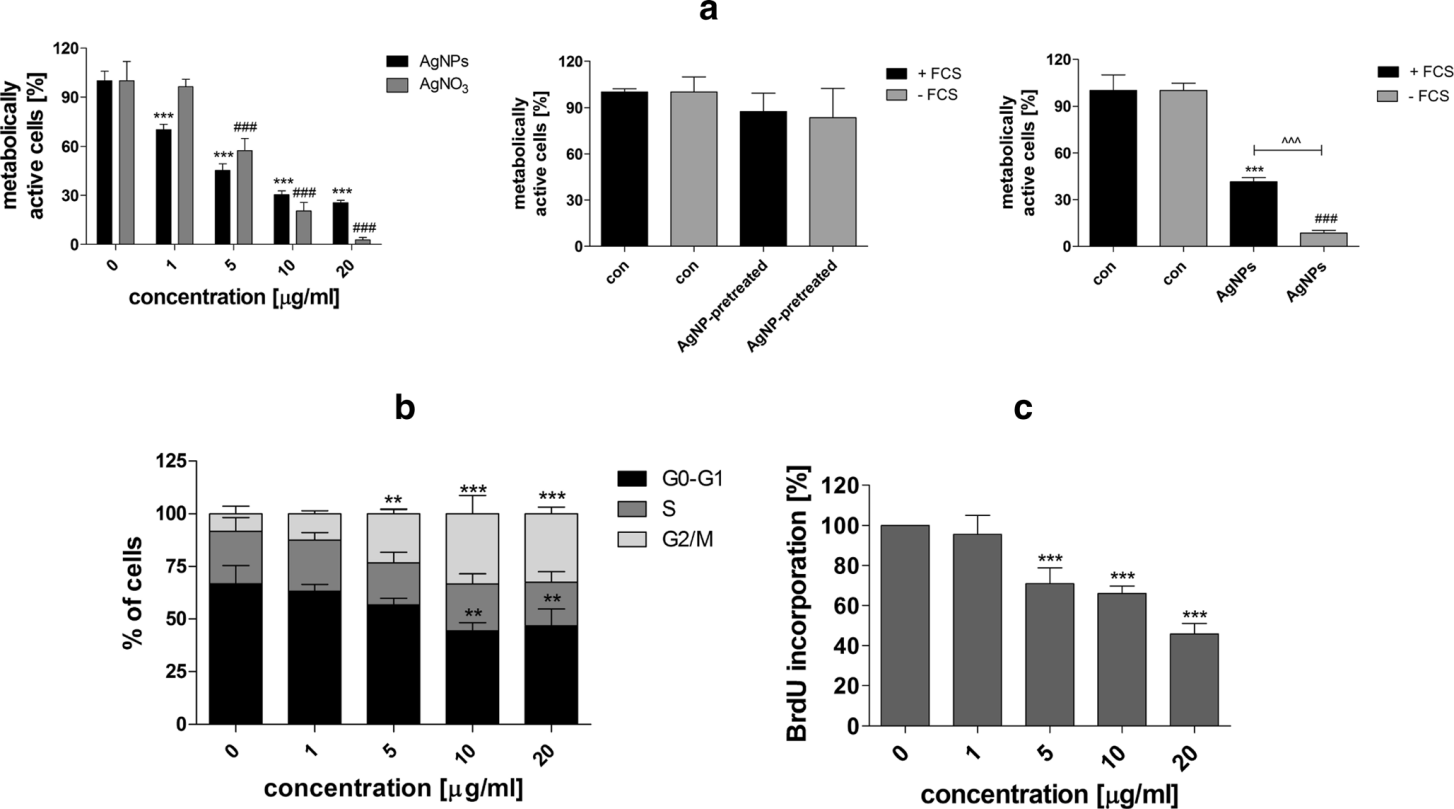
AgNP-induced cell viability (**a**), changes in the cell cycle (**b**), and cell proliferation (**c**). HT22 cells were treated with AgNPs (1–20 μg/ml) for 48 h. **a** MTT assay. Metabolic activity at standard growth conditions is considered as 100 %. The *bars* indicate the SD, *n* = 5, ***p < 0.001 compared to control (ANOVA and Dunnett’s a posteriori test). HT22 cells were also treated with AgNO_3_ (1–20 μg/ml) for 48 h for comparison (*left*). ^###^ *p* < 0.001 compared to control (ANOVA and Dunnett’s *a posteriori* test). The effect of silver ion release from AgNPs was also evaluated (*middle*). DMEM medium was pre-incubated with 5 μg/ml AgNPs for 48 h and supernatant was used as a “normal” culture medium; con, culture in DMEM with and without serum; AgNP-pretreated, culture in AgNP-pretreated DMEM with and without serum. The effect of serum on AgNP-mediated cell viability (*right*). HT22 cells were incubated with 5 μg/ml AgNPs in the presence or the absence of serum in DMEM medium for 48 h. ***p < 0.001 compared to control culture in complete medium, ^###^ *p* < 0.001 compared to control culture in a serum-free medium, ^^^*p* < 0.001 compared to 5 μg/ml AgNP treatment in complete medium (ANOVA and Dunnett’s a posteriori test). **b** Cell cycle analysis using imaging cytometry (In Cell Analyzer 2000, GE Healthcare, UK) and ImageJ software. The *bars* indicate the SD, *n* = 200, ***p < 0.001, **p < 0.01, compared to control (ANOVA and Dunnett’s a posteriori test). **c** Cell proliferation was estimated as the ability of cells to synthesize DNA: BrdU incorporation assay. Cell proliferation at standard growth conditions is considered as 100 %. The *bars* indicate the SD, *n* = 200, ***p < 0.001 compared to control (ANOVA and Dunnett’s a posteriori test)

HT22 mouse hippocampal cells were then subjected to AgNP treatment (1–20 μg/ml) for 48 h, and AgNP-mediated cytotoxicity, changes in the cell cycle, and cell proliferation were evaluated (Fig. 2).

AgNPs caused a decrease in the number of metabolically active cells (MTT assay; Fig. 2a, left). The effect was concentration-dependent and statistically significant (*p* < 0.001; Fig. 2a). The IC_50_ value was estimated to be 5 μg/ml (Fig. 2a, left). The toxicity of AgNO_3_ was also considered (Fig. 2a, left). At lower concentrations (up to 5 μg/ml), AgNO_3_ was found to be less toxic than AgNPs (Fig. 2a, left) that may suggest that AgNP effect differs from AgNO_3_ effect on HT22 cells. To further characterize the involvement of silver ions in AgNP toxicity, DMEM culture medium (with and without serum) was pretreated with AgNPs for 48 h to allow for silver ion release to the culture medium, centrifuged, and used as a “normal” culture medium. We were unable to observe any significant differences between pretreatment and control conditions (Fig. 2a, middle).

The involvement of serum was also assayed (Fig. 2a, right). The toxicity of 5 μg/ml AgNPs was potentiated in a serum-free medium (*p* < 0.001; Fig. 2a, right) that may be a result of protein corona formation-mediated effects on the uptake, fate, and toxicity of AgNPs [37, 38].

AgNPs also promoted changes in the cell cycle (Fig. 2b). After AgNP treatment, cells preferentially stayed in the G2/M phase of the cell cycle. The percentage of cells in the G2/M phase of the cell cycle increased from 8 % (control conditions) to approximately 33 % (treatment with 10 μg/ml AgNPs) (*p* < 0.001), and the level of cells in the G0-G1 phase of the cell cycle decreased from 67 % (control conditions) to approximately 44 % (treatment with 10 μg/ml AgNPs; *p* < 0.01; Fig. 2b). Moreover, the percentage of cells in the S phase of the cell cycle slightly dropped from 25 % (control conditions) to approximately 22 % (treatment with 10 μg/ml AgNPs; Fig. 2b). However, the effect was not statistically significant. AgNPs also affected cell proliferation (Fig. 2c). After 5 and 20 μg/ml AgNP treatment, 30 and 55 % of cells were unable to incorporate BrdU into their DNA, respectively, compared to control (*p* < 0.001; Fig. 2c).

**Fig. 3:**
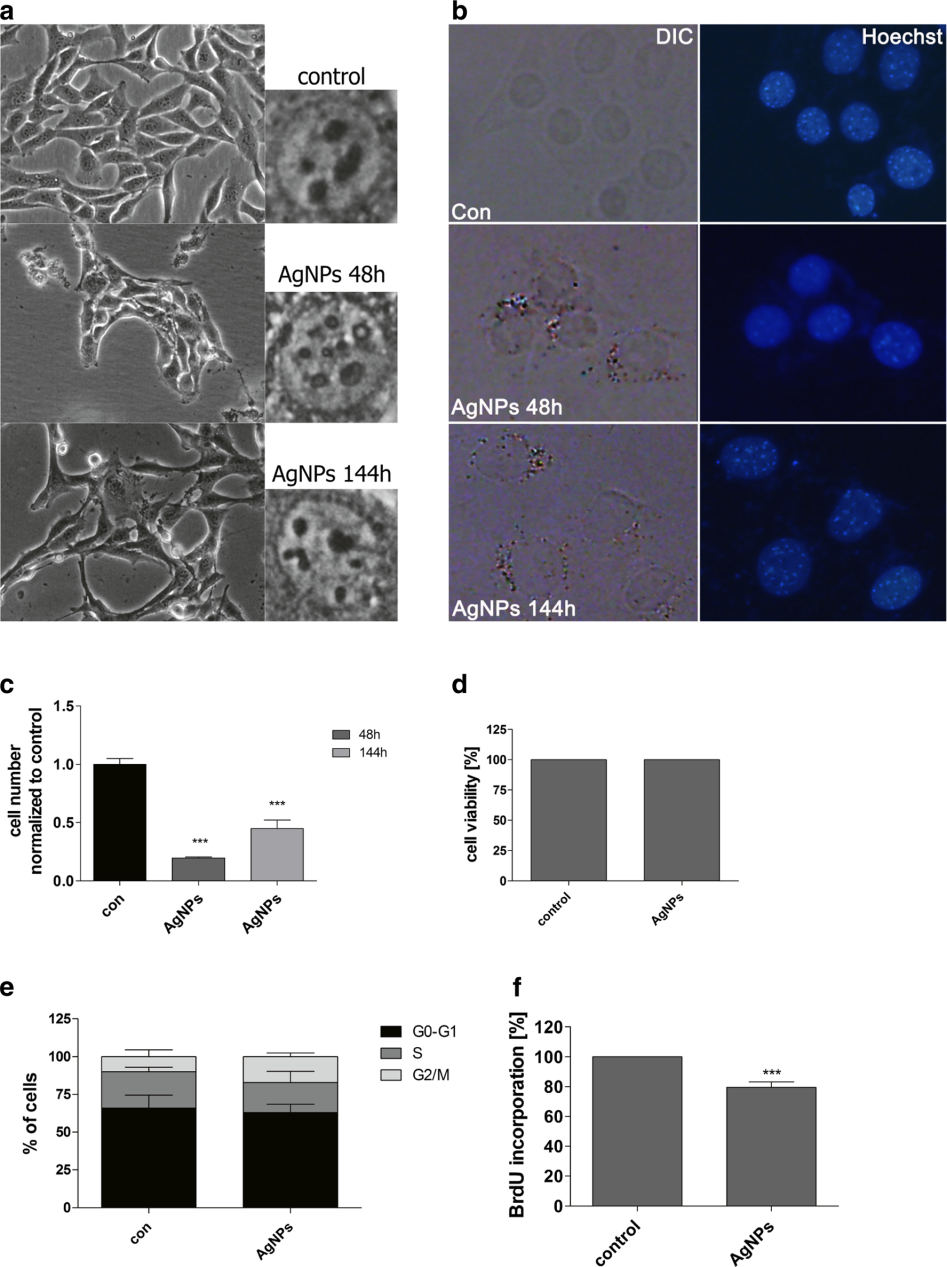
Impact of 5 μg/ml AgNP treatment and removal on cell morphology (**a**), cellular localization of AgNPs (**b**), cell number (**c**), cytotoxicity (**d**), cell cycle (**e**), and cell proliferation (**f**). HT22 cells were treated with 5 μg/ml AgNPs for 48 h, AgNPs were then removed and cells were cultured for another 96 h (a total culture time was 144 h). **a** Cells were inspected using inverted microscope. **b** AgNPs were visualized using differential interference contrast (DIC) microscopy and DNA was stained using Hoechst staining (*blue*). **c** Cell number was assessed using a Bürker chamber. The *bars* indicate the SD, *n* = 3, ***p < 0.001 compared to control (ANOVA and Dunnett’s a posteriori test). **d** Cell viability was assessed using acridine orange-ethidium bromide staining. **e** DNA-content-based cell cycle analysis using imaging cytometry (In Cell Analyzer 2000, GE Healthcare, UK) and ImageJ software. The *bars* indicate the SD, *n* = 200. **f** Cell proliferation was estimated as the ability of cells to synthesize DNA: BrdU incorporation assay. Cell proliferation at standard growth conditions is considered as 100 %. The bars indicate the SD, *n* = 200, ***p < 0.001 compared to control (ANOVA and Dunnett’s a posteriori test) (color figure online)

We then asked the question of whether AgNP effects may be prolonged and may affect cytophysiology of HT22 cells after AgNP removal. A concentration reflecting the IC_50_ value (MTT assay), 5 μg/ml, was selected for further analyses. HT22 cells were incubated with 5 μg/ml AgNPs for 48 h, washed, and cultured in AgNP-free medium for up to 144 h (96 h after AgNP removal), and morphology, AgNP localization, cytotoxicity, cell cycle, and cell proliferation analyses were conducted (Fig. 3).

First, HT22 cell morphology was compared after 48-h treatment and AgNP removal (Fig. 3a). AgNP treatment affected cell morphology (Fig. 3a). The cells formed clusters and the number of nucleoli was increased (Fig. 3a), whereas AgNP removal resulted in cell morphology more similar to control conditions (Fig. 3a). Intracellular localization (uptake) of AgNPs was confirmed using Nomarski microscopy (Fig. 3b). After AgNP removal, AgNPs were also observed in HT22 cells (Fig. 3b). After 5 μg/ml AgNP removal, the number of cells was decreased compared to control (*p* < 0.001; Fig. 3c). However, when considering 48-h treatment, the cell yield was slightly increased (Fig. 3c). After 5 μg/ml AgNP removal, cytotoxic effects were not observed (Fig. 3d). In contrast, changes in the cell cycle and the inhibition of cell proliferation were noticed (Fig. 3e, f). The percentage of cells in the G2/M phase of the cell cycle increased from 10 % (control conditions) to approximately 17 % (after AgNP removal; Fig. 3e). Moreover, the percentage of cells in the S phase of the cell cycle dropped from 24 % (control conditions) to approximately 20 % (after AgNP removal; Fig. 3e). However, the effects were not statistically significant. After 5 μg/ml AgNP removal, 20 % of cells were unable to incorporate BrdU into their DNA compared to control (*p* < 0.001; Fig. 3f).

As AgNPs may still modulate HT22 cell cycle and proliferation after their removal, we decided to evaluate if AgNP removal may also affect intracellular redox homeostasis, 53BP1 recruitment, and the levels of p53, p21, and lamin B1, and affect methylation parameters and RNA status.

**Fig. 4:**
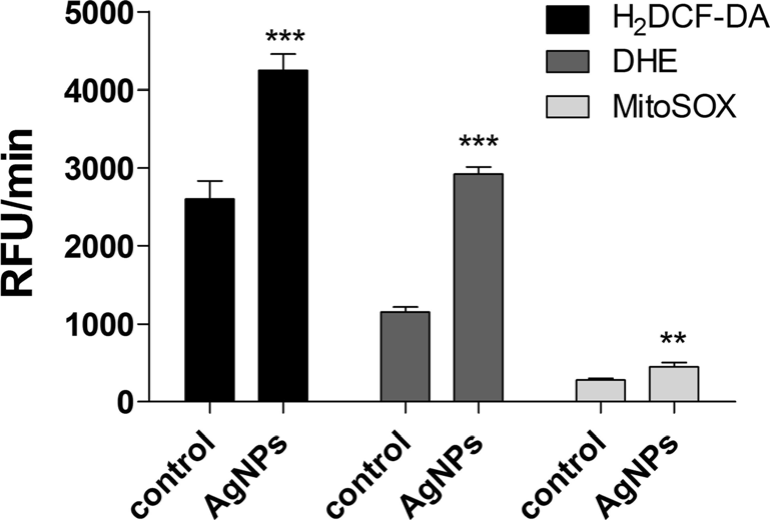
AgNP-induced oxidative stress. HT22 cells were treated with 5 μg/ml AgNPs for 48 h, AgNPs were then removed, and cells were cultured for another 96 h (a total culture time was 144 h). Total reactive oxygen species (ROS) production, intracellular superoxide production both total and mitochondrial were measured with 2′,7′-dichlorodihydrofluorescein diacetate (H_2_DCF-DA), dihydroethidium, and MitoSOX^TM^, respectively. Fluorescence intensity was monitored in a Tecan Infinite® M200 fluorescence mode microplate reader. The *bars* indicate the SD, *n* = 5, ***p < 0.001, **p < 0.01 compared to control (ANOVA and Dunnett’s a posteriori test)

The removal of 5 μg/ml AgNPs was accompanied by oxidative stress (Fig. 4).

A 1.6-, 2.5-, and 1.6-fold increase in intracellular ROS, total superoxide, and mitochondrial superoxide production was observed, respectively, compared to control (*p* < 0.01 and *p* < 0.001; Fig. 4).

**Fig. 5:**
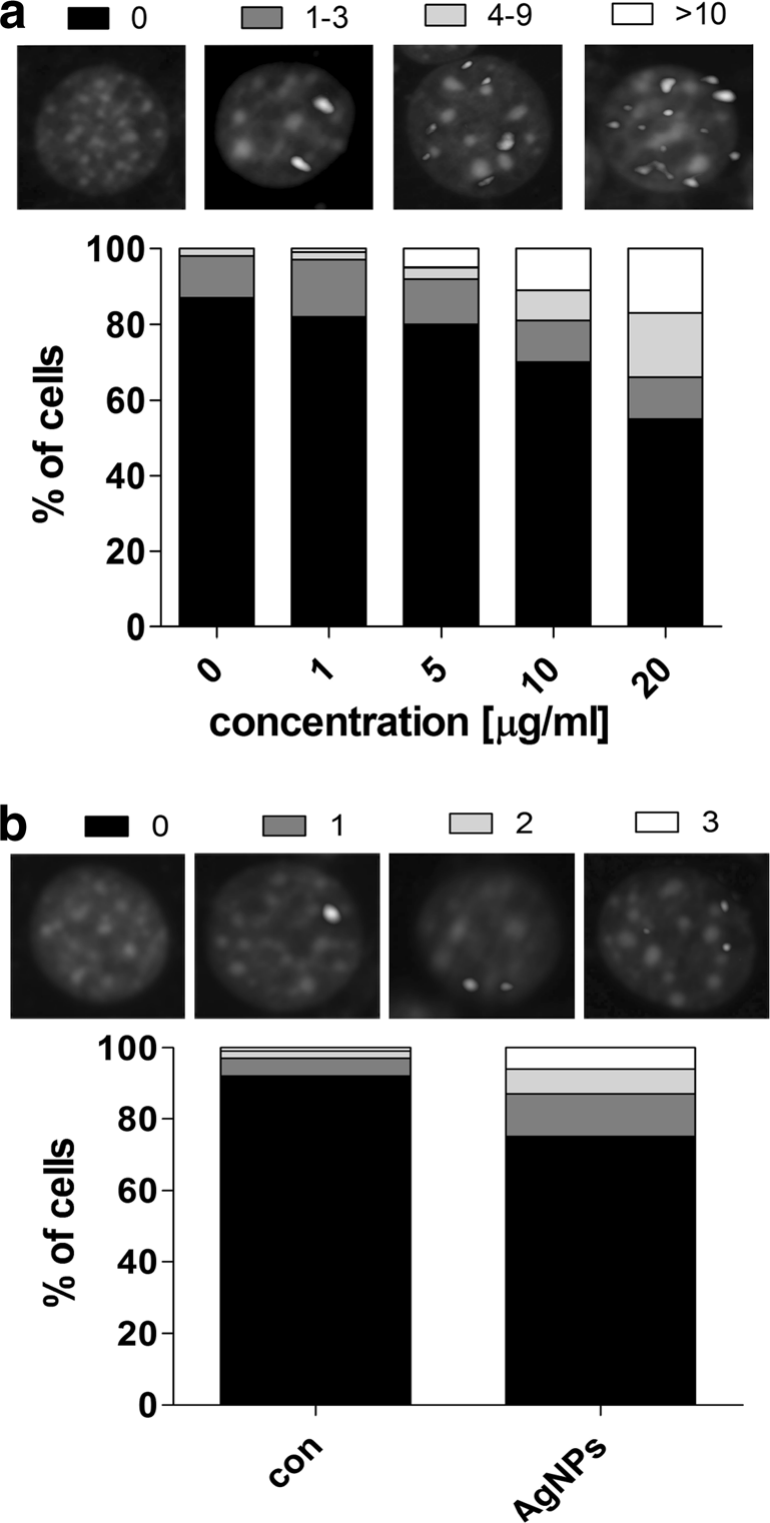
AgNP-mediated 53BP1 recruitment. HT22 cells were treated with AgNPs (1–20 μg/ml) for 48 h (**a**) or HT22 cells were treated with 5 μg/ml AgNPs for 48 h and AgNPs were then removed and cells were cultured for another 96 h (a total culture time was 144 h) (**b**). 53BP1 foci were revealed using 53BP1 immunostaining. Cells with 0, 1–3, 4–9, and more than 10 53BP1 foci (**a**) or cells with 0, 1, 2 and 3 53BP1 foci (**b**) were scored (%)

We also investigated AgNP-mediated formation of p53 binding protein (53BP1) foci, which are considered to be accumulated at site of double-strand breaks (DSBs) being a part of DNA repair process. The recruitment of 53BP1 was much more accented after 48-h treatment with AgNPs (1–20 μg/ml) than after removal of 5 μg/ml AgNPs (Fig. 5).

After 10 and 20 μg/ml AgNP treatment, the level of 53BP1 foci-positive cells were 30 and 50 %, respectively, compared to 15 % level of control (Fig. 5a). However, the recruitment of 53BP1 after removal of 5 μg/ml AgNPs was also observed (Fig. 5b). The level of 53BP1 foci-positive cells increased threefold compared to untreated control (Fig. 5b).

**Fig. 6:**
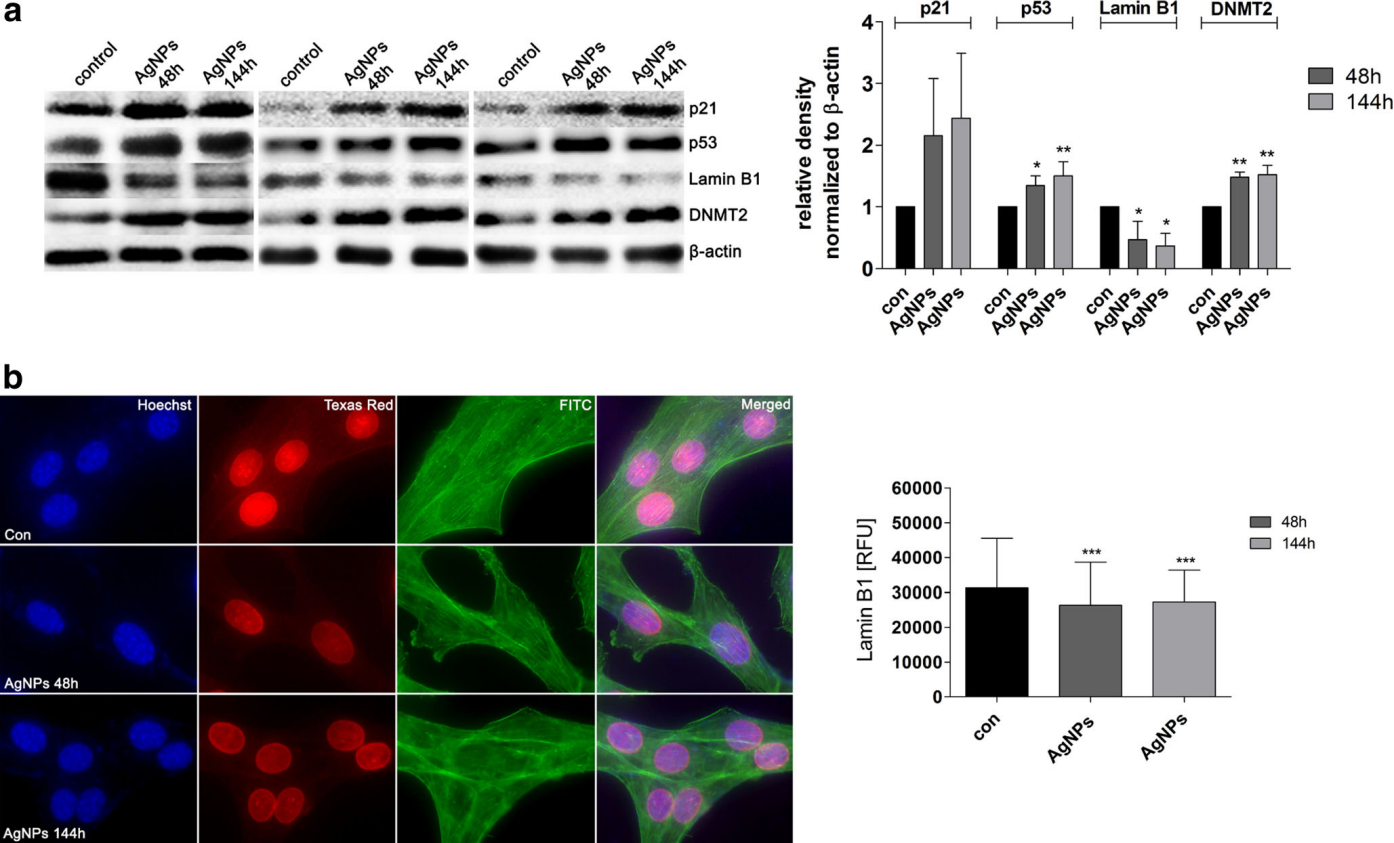
AgNP-associated levels of p21, p53, lamin B1, and DNMT2. HT22 cells were treated with 5 μg/ml AgNPs for 48 h or HT22 cells were treated with 5 μg/ml AgNPs for 48 h and AgNPs were then removed and cells were cultured for another 96 h (a total culture time was 144 h). **a** Western blot analysis of p21, p53, lamin B1, DNMT2, and β-actin levels. Three blots representing three independent experiments are shown. The *bars* indicate the SD, *n* = 3, **p < 0.01, *p < 0.05 compared to 48 h control (ANOVA and Dunnett’s a posteriori test). The data represent the relative density normalized to β-actin. **b** Immunofluorescence-based analysis of lamin B1 level (*red*). DNA was stained using Hoechst 33342 staining (*blue*). F-actin was labeled using Alexa Fluor® 488 Phalloidin staining (*green*). Lamin B1 nuclear signals are presented as relative fluorescence units (RFUs). The *bars* indicate the SD, *n* = 2000, ***p < 0.001 compared to 48 h control (ANOVA and Dunnett’s a posteriori test) (color figure online)

After AgNP treatment, the levels of p53 and p21 were elevated (Fig. 6a), which may contribute to AgNP-mediated antiproliferative activity (Fig. 2).

Moreover, the levels of p53 and p21 remained high after AgNP removal (Fig. 6a).

AgNPs also caused a diminution in lamin B1 pools (Fig. 6a, b) that was not accompanied by changes in the nucleus structure or F-actin cytoskeleton (Fig. 6b).

**Fig. 7:**
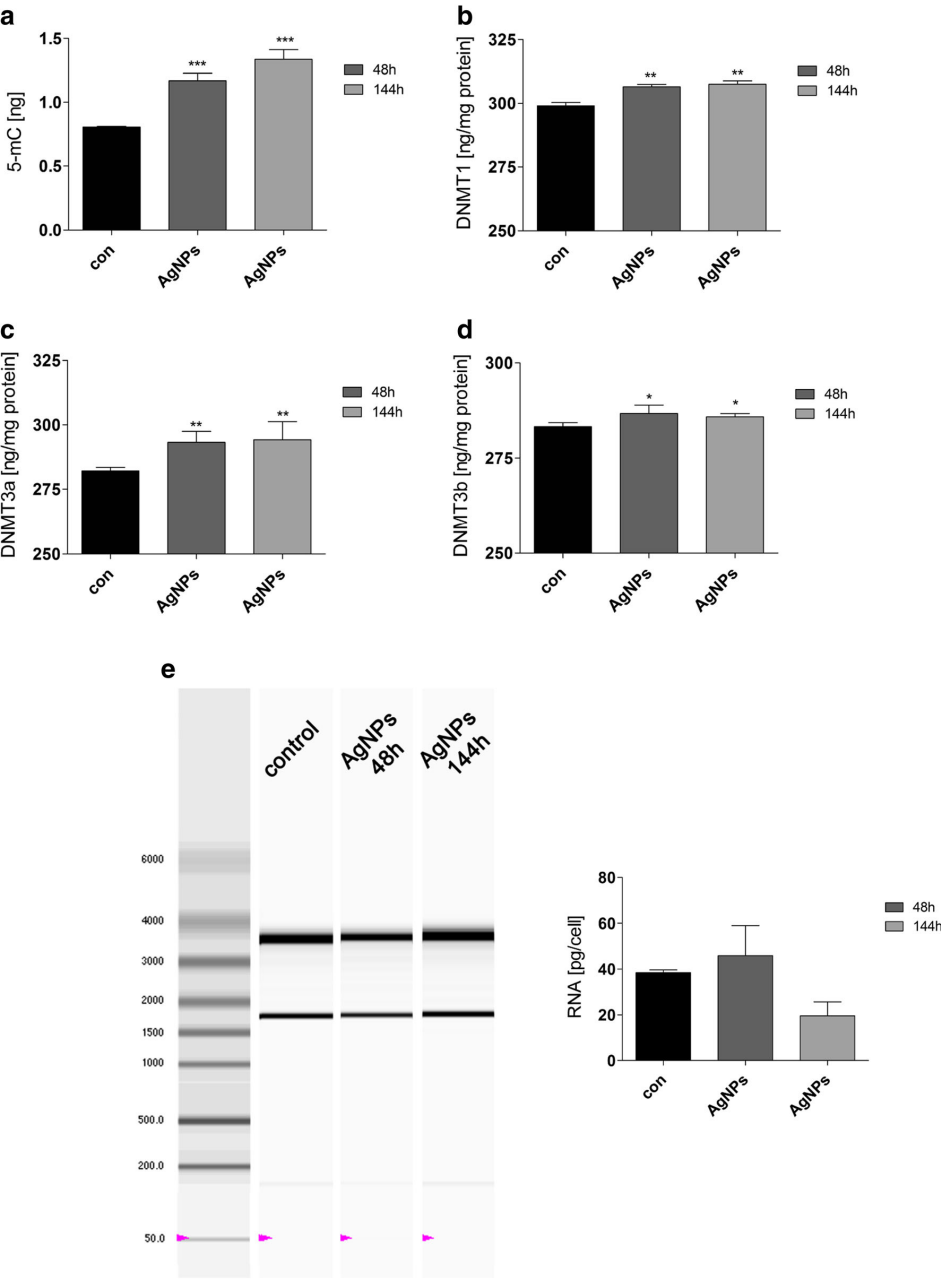
AgNP-induced changes in methylation parameters and RNA status. HT22 cells were treated with 5 μg/ml AgNPs for 48 h or HT22 cells were treated with 5 μg/ml AgNPs for 48 h and AgNPs were then removed and cells were cultured for another 96 h (a total culture time was 144 h). **a** 5-Methylcytosine (5-mC) level (ng) (ELISA), **b** DNMT1 level (ng/mg protein) (ELISA), **c** DNMT3a level (ng/mg protein) (ELISA), and **d** DNMT3b level (ng/mg protein) (ELISA). The *bars* indicate the SD, *n* = 3, ***p < 0.001, **p < 0.01, *p < 0.05 compared to 48 h control (ANOVA and Dunnett’s a posteriori test). **e** RNA chip electrophoresis. RNA molecular weight marker is also shown. Total RNA (pg) is calculated per cell. The *bars* indicate the SD, *n* = 3

Finally, AgNPs stimulated methylation changes (Figs. 6 and 7). The effects were observed both after 48-h treatment with AgNPs and after AgNP removal (Figs. 6 and 7).

AgNPs can be considered as a DNA hypermethylating agent in HT22 cells, because AgNPs induced an increase in the levels of 5-methylcytosine (5-mC), and DNA methyltransferases 1, 3a, and 3b (DNMT1, DNMT3a, and DNMT3b; Fig. 7a–d). After AgNP removal, the content of 5-mC was increased approximately 50 % compared to control (*p* < 0.001; Fig. 7a). After AgNP removal, an increase in DNMT1, DNMT3a, and DNMT3b levels was less impressive compared to increased 5-mC levels, but these effects were statistically significant (*p* < 0.01 and *p* < 0.05; Fig. 7b–d).

Surprisingly, after AgNP removal, the level of DNMT2, a methyltransferase suggested to be involved in cellular stress responses, was elevated approximately 50 % compared to control (Fig. 6a). As DNMT2 may have a protective role against RNA degradation [32], we evaluated RNA status after AgNP treatment and AgNP removal (Fig. 7e). The ratio of 28S/18S rRNA was unaffected (values between 1.8 and 2.0) that may provide evidence that RNA integrity (quality) was not compromised upon AgNP stimulation (data not shown). AgNP treatment did not result in changes in RNA pools, but after AgNP removal, RNA synthesis was diminished (Fig. 7e) that may reflect decreased transcriptional activity of AgNP-treated cells as a result of increased levels of DNMTs and subsequent global DNA hypermethylation (Fig. 7a-d).

## Discussion

AgNP-mediated response in HT22 hippocampal neuronal cells was investigated; especially, attention was paid on low concentration (5 μg/ml) and prolonged effects of AgNPs. We showed for the first time that AgNP-induced effects retained after AgNP removal from the cell culture medium. AgNPs affected cell cycle, proliferation, redox homeostasis, response to DNA damage, and the levels of p53, p21, and lamin B1, and promoted methylation changes that affected RNA synthesis.

AgNP toxicity is well documented [4–7]. It has been postulated and rebutted that AgNP toxicity and related biological effects may be due to silver ion toxicity [12, 14–17, 21]. In the present study, the effect of silver ion release from AgNPs was ruled out as there were no effects of AgNP-pretreated medium supernatant on HT22 cells. We found that AgNP toxicity may be affected by the presence of serum in the cell culture medium, namely, in a serum-free medium AgNP toxicity may be potentiated. Perhaps, AgNP toxicity may be modulated by the formation of a protein corona (PC) on its surface (this study). The fractions of enriched serum proteins were observed when cell culture medium was concentrated and subjected to protein corona analysis. Indeed, addition of the PC decreased uptake of AgNPs by rat lung epithelial and rat aortic endothelial cells and affected cellular toxicity *via* scavenger receptors [39]. Additionally, nanoparticle agglomeration in the cell culture medium may affect its toxic activities [40–43]. However, we did not observe AgNP tendency to agglomerate in the cell culture medium using AFM imaging.

The mechanisms of AgNP toxicity involve oxidative stress, DNA damage, and apoptosis that have been shown in numerous human cell lines in vitro [1, 4, 5, 8–14]. AgNP-mediated neurotoxicity has also been considered [22–26], especially that nanoparticles may not only cause adverse effects in primary organs directly exposed but also in secondary organs, such as the central nervous system (CNS).

AgNPs affected intracellular redox homeostasis by increasing reactive oxygen species (ROS) production, lipid peroxidation, and protein carbonylation, and/or decreasing the levels of reduced glutathione and the activity of antioxidant enzymes (e.g., superoxide dismutase and catalase) [4, 5, 8–14]. AgNP-mediated oxidative stress and calcium dysregulation have also been reported in neuronal cells that may promote apoptotic cell death and/or neurodegeneration [22, 23, 26]. AgNP-induced apoptosis involved mitochondrial pathway as AgNP treatment resulted in cytochrome *c* release into the cytoplasm and translocation of Bax to mitochondria in NIH3T3 fibroblast cells and human Chang liver cells [10, 13]. Moreover, AgNPs may promote alterations in the mitochondrial membrane potential (MMP) leading to abnormalities in physiological functions of mitochondria, which are common during stress-induced apoptotic cell death [11, 13]. AgNP-induced oxidative stress may also stimulate genotoxic events. Indeed, AgNP treatment resulted in DNA adducts, DNA breaks, oxidative DNA damage (increased 8-oxoguanine level), and micronuclei production [4, 13, 44–47]. AgNPs also decreased 8-oxoguanine DNA glycosylase 1 (OGG1, DNA repair enzyme that recognizes and excises 8-oxoguanine) mRNA and protein expression, resulting in decreased OGG1 activity [44]. Thus, decreased OGG1 activity in AgNP-treated cells led to increased 8-oxoguanine levels [44]. Surprisingly, HT22 cells manifested DNA damage response (53BP1 recruitment) even after AgNP removal from the cell culture medium, which suggest that AgNPs may also promote genotoxic effects (e.g., DNA double strand breaks) in these cells. Under DNA double-strand break (DSB)-promoting conditions, a complex cellular response is activated, which enables to promote DNA repair and maintain genome integrity [48], and 53BP1, p53 binding protein, is recruited to sites of DNA damage due to methylation-state-specific recognition of histone H4-K20 by 53BP1 [49]. Perhaps elevated levels of p53 and p21 are a part of HT22 cell response to DNA damage promoting conditions (AgNP treatment) and may account for observed antiproliferative activity of AgNPs in these cells to allow time for DNA repair.

AgNPs also decreased the levels of lamin B1, but this did not significantly affect the structure of nucleus of a HT22 cell. We have previously shown that diamond, silica, and silver nanoparticles may promote a diminution in lamin B1 pools in different cell lines both normal and cancer cells that is a part of telomere-focused adaptive response [36]. More recently, the effects of cobalt chrome (CoCr) nanoparticles on nuclear morphology in human fibroblasts were studied [50]. Nano-CoCr treatment resulted in oxidative-stress-mediated loss of mature lamin B1 [50]. Mitochondrial ROS were implicated in damage to lamin B1, increased incidence of micronuclei, and misshapen nuclei [50]. Downregulation of lamin B1 may also modulate the expression of antioxidant proteins and subsequent gene expression either through p53 or Oct-1 [51, 52]. Moreover, nuclear lamins as a ROS sink were suggested to be mediators of oxidative stress [53].

As actin cytoskeleton may be disrupted by the action of sublethal concentrations of AgNPs (0.1–1 μg/ml) in cultured adult neural stem cells [27], we decided to evaluate if 5 μg/ml AgNP treatment and then AgNP removal may modulate actin cytoskeleton dynamics in HT22 cells. F-actin cytoskeleton was unaffected upon AgNP stimulation (this study). At higher concentration used (up to 50 μg/ml), AgNPs disrupted filamentous actin, β-tubulin, and synaptic machinery in cultured cortical neurons [54]. The authors also concluded that associated disruption in neurogenesis may contribute to documented deficits in brain function following AgNP exposure [27, 54].

Epigenetics, heritable modifications that alter gene expression levels without resulting from direct changes in the primary DNA sequence, is implicated in both physiological and pathophysiological processes, such as development, cell proliferation and differentiation, genetic imprinting, X chromosome inactivation, maintenance of chromatin structure, tumor progression, cellular senescence, and organism aging [55–59]. In mammals, the main epigenetic mechanisms for gene regulation are DNA methylation, histone tail modifications (acetylation, phosphorylation, methylation), and microRNA (miRNA)-mediated mechanisms [60, 61]. Gene silencing is a result of DNA hypermethylation, whereas DNA hypomethylation activates transcription. DNA methyltransferases (DNMTs) catalyze the transfer of a methyl group from S-adenosylmethionine (SAM) to cytosine within CpG sequences to form 5-methylcytosine (5-mC) [62]. It is widely accepted that DNMT1 is involved in the maintenance of DNA methylation patterns during development and cell division, whereas DNMT3a and DNMT3b are the de novo methyltransferases [63, 64]. The role of DNMT2 is more enigmatic, but it may participate in the methylation of transfer RNA molecules [65]. We showed that AgNPs may affect HT22 cell epigenome by increasing the levels of 5-mC, DNMT1, DNMT3a, and DNMT3b and acting as a DNA hypermethylating agent. Data on epigenetic properties of engineered nanomaterials, especially DNA-methylation-based effects, are limited [66], and published results on AgNP-mediated changes in DNA methylation patterns are unavailable. There are two papers on nano-SiO_2_-induced changes in global and *loci*-specific DNA methylation [67, 68]. Nano-SiO_2_ promoted global DNA hypomethylation (a decrease in 5-mC level), which was accompanied by decreased DNMT1 and DNMT3a mRNA and protein levels in HaCaT cells [67]. Moreover, nano-SiO_2_ caused *PARP-1* hypermethylation and *PARP-1* mRNA repression affecting DNA damage repair process in HaCaT cell line [68]. In contrast, AgNPs were found to be a modulator of microRNA profiles in Jurkat T cells [21]. An integrated analysis of mRNA and miRNA expression revealed that the expression of hsa-miR-219-5p was negatively correlated with the expression of metallothionein 1 F (MT1F) and tribbles homolog 3 (TRIB3), and epigenetic mechanism was suggested to be involved in the toxicity of AgNPs in Jurkat T cells [21].

AgNPs also induced DNMT2 protein expression, which may be considered as a part of stress response. The role of DNMT2 in both DNA methylation and RNA methylation has been proposed [64, 69, 70]. DNA methyltransferase activity of human DNMT2 and *Drosophila* Dnmt2 has been reported [71, 72]. Moreover, a highly specific tRNA^Asp^ methyltransferase activity of DNMT2 has been postulated and rebutted [65, 73]. Regardless of the mechanism involved, DNMT2 is implicated in the protection against cellular stresses, especially oxidative stress, in different biological systems [74–76]. More recently, DNMT2 was found to be upregulated in HeLa cells upon treatment with nanodiamonds, which contributed to RNA stabilization and conferred stress resistance after nanodiamond treatment [32]. Both stimulations—nanodiamond [32] and AgNP (this study) treatments—resulted in oxidative stress in HeLa and HT22 cells, respectively, and response to imbalanced redox homeostasis may involve DNMT2-based protective response against RNA degradation. Indeed, after AgNP treatment, RNA integrity was not compromised.

In conclusion, we showed for the first time that AgNPs may modulate HT22 cell proliferation, DNA damage response, and epigenome acting as a DNA hypermethylating agent. Thus, AgNPs may promote epigenetic dysregulation, which may have long-term effects on gene expression re-programming. Moreover, AgNP-induced effects may also be manifested at the epigenomic level. As human exposure to nanomaterials is rapidly increasing, it seems worthwhile to study in detail the subsequent physiological effects of AgNP-mediated epigenetic changes in biological systems including neuronal cells and tissue.
